# Multi-Organ Alcohol-Related Damage: Mechanisms and Treatment

**DOI:** 10.3390/biom6020020

**Published:** 2016-04-15

**Authors:** Natalia A. Osna, Kusum K. Kharbanda

**Affiliations:** 1 Research Service, Veterans Affairs Nebraska-Western Iowa Health Care System, Omaha, NE 68105, USA; 2 Department of Internal Medicine, University of Nebraska Medical Center, Omaha, NE 68105, USA; 3 Department of Biochemistry and Molecular Biology, University of Nebraska Medical Center, Omaha, NE 68198, USA

Alcohol consumption causes damage to various organs and systems. Liver is a primary target for the detrimental effects of alcohol since this substance of abuse is mainly metabolized by liver cells, which express high levels of two major alcohol oxidizing enzymes, alcohol dehydrogenase and CYP2E1. However, other organs, including brain, gut, pancreas, lungs and the immune system are also affected by alcohol. Alcohol may also serve to intensify the progression of viral infections, autoimmune diseases and cancer. Common mechanisms of alcohol-related organ injury include increases in oxidative stress, methylation impairments, aberrant posttranslational modifications of proteins, dysregulation in lipid metabolism and signal transduction pathways, all of which ultimately affect cell survival and function.

The Special Issue on “Multi-Organ Alcohol-Related Damage: Mechanisms and Treatment” presents 17 review and 9 original articles which cover diverse topics related to the pathogenesis of organ dysfunction by alcohol exposure. Here, we provide a short summary of all the articles published in this Special Issue.

Several articles address **common mechanisms** of multi-organ alcohol-induced organ damage. In this regard, the paper by Natarajan *et al.* [1] discusses the role of microRNAs in the pathogenesis of alcohol-induced pancreatitis, liver damage, cardiomyopathy, muscle damage, intestinal epithelial barrier dysfunction and brain damage, particularly the altered hippocampus structure and function and neuronal loss. The review by Anji and Kumari [2] focusses on the effect of alcohol on different RNA-binding proteins and their possible contribution to alcohol-related disorders. The authors present a thoughtful deliberation on the role of these proteins in the development of neurological diseases and cancer as well as the conventional methods and newer techniques that are employed to identify RNA-binding proteins. The review by Steiner *et al.* [3] discusses the glucose metabolic effects of alcohol in the muscle, liver and adipose tissue under basal post-absorptive conditions and in response to insulin stimulation. They present evidence that alcohol intake is related to the development and/or exacerbation of type 2 diabetes and demonstrate the necessity of employing a multi-systems approach since both alcohol and diabetes affect multiple targets within the body. Ji [4] presents the recent advances in alcohol-induced organelle stress, unfolded protein responses and organ damage. His review describes the alcohol-altered proteostasis, endoplasmic reticulum stress response as well as discusses new concepts on alcohol-induced mitochondrial, Golgi and lysosomal stress responses in the pathogenesis of tissue injury. 

Since liver is a primary site of ethanol metabolism and liver cells are susceptible to alcohol-induced damage, several articles in this special issue are on the effects of alcohol in promoting liver injury. Among them is the study by Shukla *et al.* [5] that focuses on the epigenetic changes induced by acute and chronic ethanol consumption. They clearly demonstrate that ethanol ingestion elicit characteristic profiles of histone modifications, metabolic alterations and changes in nuclear protein levels that accelerates the progression of alcoholic liver disease. A timely review is presented by Kirpich *et al.* [6] that provides the current understanding of recent advances regarding the role of dietary lipids in alcoholic liver disease pathogenesis. The review by Groebner and Tuma [7] hypothesize that α-tubulin is a major target for modification by highly reactive ethanol metabolites and reactive oxygen species. They discuss the potential cellular consequences of microtubule modification in promoting hepatic dysfunction, with a focus on the alcohol-induced defects in protein trafficking and enhanced steatosis. Two articles are devoted to autophagy and mitophagy as important protective mechanisms in alcoholic liver disease. One, an original article by Lu and Cederbaum [8] presents elegant data obtained employing an ethanol fed mouse model system. Their study demonstrates that autophagy is protective against CYP2E1-dependent liver injury by minimizing the ethanol-induced CYP2E1-dependent oxidative stress and the development of steatosis and liver injury. This indicates that attempts to stimulate autophagy may be helpful in lowering ethanol and CYP2E1-dependent liver toxicity. The second, an article by Williams and Ding [9] presents an overview on the protective role of mitophagy in preventing alcohol-induced liver damage. Specifically, they discuss the roles of both Parkin-dependent and -independent mechanisms of mitophagy activation in protection against alcohol-induced liver injury and steatosis.

Udoh *et al.’s* review article [10] on the role of impairment in the circadian clock in alcoholic liver disease development proposes that the alcohol-mediated disruption in circadian rhythms likely underpins many adverse health effects of alcohol that cut across multiple organ systems. The authors summarize various molecular events by which alcohol may negatively impact circadian clock-mediated processes in the liver, thereby contributing to tissue pathology. The article by Szary *et al.* [11] devoted to high intrinsic endurance/aerobic capacity presents a connection between high aerobic fitness and protection from metabolic diseases. This study, using a high capacity runner rat model, demonstrates that ethanol ingestion fails to induce significant hepatic liver injury when different parameters such as hepatic inflammation or serum alanine amino transferase, free fatty acids, triglycerides, insulin and glucose levels were measured. While high intrinsic aerobic fitness protected against ethanol-induced hepatic injury and systemic metabolic dysfunction, it did not reduce ethanol-activated hepatic steatosis. The review by Neuman *et al.* [12] elucidates the mechanisms by which alcohol contributes to the activation of Kupffer cells and the inflammatory cascade and deliberates the role of the stellate cells in fibrogenesis. Another article by Nanau and Neuman [13] addresses the diagnostic value of biomolecules and biomarkers in alcohol drinking, especially in monitoring therapeutic interventions. The study by Harrison-Findik and Lu [14] investigates the regulation of the key iron-regulatory molecule, hepcidin, by alcohol and hydrogen peroxide (H_2_O_2_). Employing glutathione peroxidase-1 (gpx-1^−/−^) and catalase (catalase^−/−^) knockout mice, the authors conclude that H_2_O_2_ inhibits hepcidin expression *in vivo* and that synergistic induction of CCAAT-enhancer-binding protein homologous protein (CHOP) by alcohol and H_2_O_2_, in the absence of gpx-1, stimulates liver hepcidin gene expression by endoplasmic reticulum stress independent of CREBH. The review “Update on Alcoholic Hepatitis” by Torok [15] presents recent advances in the diagnosis, pathogenesis of alcoholic hepatitis and novel treatment strategies. The study by Scheer *et al.* [16] demonstrates that acetaldehyde has a major role in the ethanol metabolism-mediated G2/M cell cycle arrest, and the concurrent accumulation of p21 (a cyclin-dependent kinase inhibitor) and p-Cdc2 (cell divison cycle protein 2 homolog). Although reactive oxygen species are thought to have a significant role in ethanol-induced hepatocellular damage, they may have a less important role in the inability of hepatocytes to replace dead or damaged cells. An article by Deshpande *et al.* [17] highlights previously unknown effects of moderate ethanol exposure on hepatic wound healing after acute carbon tetrachloride exposure by indicating that moderate ethanol affected each phase of the wound healing response to this hepatotoxicant. Finally, the review article by Gitto *et al.* [18] discusses the usefulness of psychosocial support in the management of people affected by alcohol. These authors reflect that a multidisciplinary approach involving clinical-psychologists, psychiatrics and hepatologists should be considered as essential in the management of patients with alcohol liver disease especially in the case of liver transplantation.

Several studies/reviews present an association between the ethanol-induced **gut dysfunctions** and the pathogenesis of **liver injury**. Patel *et al.* [19] review the mechanisms of alcohol-induced endotoxemia (including dysbiosis and gut leakiness) as well as the predisposing factors that promote these changes. They also describe various barriers, including immunologic, physical, and biochemical that regulate the passage of toxins into the portal and systemic circulation. In addition, the review by Massey *et al.* [20] discusses the cross-talk between gut, liver and lung and the parallel mechanisms of liver and lung injury in response to alcohol consumption. The authors also explore the potential that these mechanisms are interdependent, as part of a gut-liver-lung axis

The study by Zhong *et al.* [21] illustrates that while alcohol consumption causes nicotinic acid deficiency, its supplementation upregulates the intestinal genes involved in aldehyde detoxification via transcriptional regulation. This indicates that the inhibitory effects of nicotinic acid on alcohol-induced endotoxemia and hepatic inflammation are via the modulation of the intestinal barrier function and bacterial endotoxin production. 

Two articles reveal the mechanism of alcohol-induced **lung** damage. One article by Sapkota and Wyatt [22] presents an overview on the formation of reactive aldehydes in the lung as a result of drinking alcohol and smoking cigarettes that react with nucleophilic targets in cells such as DNA, lipids and proteins to form both stable and unstable adducts. The review provides an insight into different reactive aldehyde adducts and their role in the pathogenesis of lung disease. The second, a review by Traphagen *et al.* [23] follows the influence of alcohol on lung injury and the role of ethanol exposure in the pathogenesis and progression of pulmonary disease.

Alcohol is also shown to affect **heart** function. The article “Alcohol and Apoptosis: Friends or Foes?” by Rodriguez *et al.* [24] is devoted to the *in vivo* effects of alcohol exposure on cardiomyocytes contraction and relaxation. The authors demonstrate that the animals on high doses of alcohol display a marked thinning of the left ventricular wall along with elevated caspase-3 activity and decreased contractility. In contrast, low alcohol is associated with increased contractility and decreased apoptosis suggesting that overall protective mechanisms are induced by low levels of alcohol exposure.

Brand *et al.* [25] contributed to this Special Issue with the hypotheses that the skin immune network may be relatively preserved in alcohol consumers and proposed that the development of skin-targeted immunizations could circumvent the immune inhibitory effects of alcohol consumption. The authors demonstrate that independent of the feeding model, ethanol ingestion inhibits delayed type hypersensitivity, lysis by cytotoxic T-lymphocytes, and antigen-specific total IgG induced by traditional systemic vaccines. They further show that skin-targeted vaccines were equally immunogenic in alcohol-exposed and non-exposed subjects suggesting that cutaneous immunization may result in more efficacious vaccination in alcohol abusing subjects.

Only one study was related to **neurotoxic** effects of alcohol. To this end, Yang and Luo [26] review the role of ethanol-induced endoplasmic reticulum stress on neurotoxicity. The authors discuss recent progress in the study of endoplasmic reticulum stress in ethanol-induced neurotoxicity and examine the interaction among endoplasmic reticulum stress, oxidative stress and autophagy in the context of ethanol neurotoxicity.

Overall, this Special Issue on “Multi-Organ Alcohol-Related Damage: Mechanisms and Treatment” covers important aspects on alcohol-induced organ damage and provides comprehensive information on the mechanisms underlying these events. The published articles are recommended for scientists and physicians involved in basic, translational and/or clinical studies on alcohol-induced injury to cells, tissues and multiple organs.

The list of articles published in Special Issue “Multi-Organ Alcohol-Related Damage: Mechanisms and Treatment” is presented in Refs. [1,2,3,4,5,6,7,8,9,10,11,12,13,14,15,16,17,18,19,20,21,22,23,24,25,26]. 
